# Limited Relationship between Cervico-Vaginal Fluid Cytokine Profiles and Cervical Shortening in Women at High Risk of Spontaneous Preterm Birth

**DOI:** 10.1371/journal.pone.0052412

**Published:** 2012-12-26

**Authors:** Manju Chandiramani, Paul T. Seed, Nicolas M. Orsi, Uma V. Ekbote, Phillip R. Bennett, Andrew H. Shennan, Rachel M. Tribe

**Affiliations:** 1 Division of Women’s Health, King's College London, London, United Kingdom; 2 Institute of Reproductive & Developmental Biology, Imperial College London, London, United Kingdom; 3 Gynaeimmunology and Oncology Group, YCR & Liz Dawn Pathology & Translational Sciences Centre, Institute of Molecular Medicine, St James's University Hospital, Leeds, United Kingdom; Hospital Clinic, University of Barcelona, Spain

## Abstract

**Objective:**

To determine the relationship between high vaginal pro-inflammatory cytokines and cervical shortening in women at high risk of spontaneous preterm labor and to assess the influence of cervical cerclage and vaginal progesterone on this relationship.

**Methods:**

This prospective longitudinal observational study assessed 112 women with at least one previous preterm delivery between 16 and 34 weeks’ gestation. Transvaginal cervical length was measured and cervico-vaginal fluid sampled every two weeks until 28 weeks. If the cervix shortened (<25 mm) before 24 weeks’ gestation, women (cases) were randomly assigned to cerclage or progesterone and sampled weekly. Cytokine concentrations were measured in a subset of cervico-vaginal fluid samples (n = 477 from 78 women) by 11-plex fluid-phase immunoassay.

**Results:**

All 11 inflammatory cytokines investigated were detected in cervico-vaginal fluid from women at high risk of preterm birth, irrespective of later cervical shortening. At less than 24 weeks’ gestation and prior to intervention, women destined to develop a short cervix (n = 36) exhibited higher cervico-vaginal concentrations than controls (n = 42) of granulocyte-macrophage colony-stimulating factor [(GM-CSF) 16.2 fold increase, confidence interval (CI) 1.8–147; p = 0.01] and monocyte chemotactic protein-1 [(MCP-1) 4.8, CI 1.0–23.0; p = 0.05]. Other cytokines were similar between cases and controls. Progesterone treatment did not suppress cytokine concentrations. Interleukin (IL)-6, IL-8, granulocyte colony-stimulating factor (G-CSF), interferon (IFN)-γ and tumour necrosis factor (TNF)-α concentrations were higher following randomization to cerclage *versus* progesterone (p<0.05). Cerclage, but not progesterone treatment, was followed by a significant increase in cervical length [mean 11.4 mm, CI 5.0–17.7; p<0.001].

**Conclusions:**

Although GM-CSF and MCP-1 cervico-vaginal fluid concentrations were raised, the majority of cervico-vaginal cytokines did not increase in association with cervical shortening. Progesterone treatment showed no significant anti-inflammation action on cytokine concentrations. Cerclage insertion was associated with an increase in the majority of inflammatory markers and cervical length.

## Introduction

Preterm birth is a major challenge facing modern obstetrics, with a global prevalence of 9.6% and over a million annual neonatal deaths [1]. Spontaneous preterm labour (SPTL), which accounts for 75% of these births, is likely to be initiated by a variety of factors [2], thereby hindering accurate identification of at-risk women. Current clinical management is also disparate [3]–[11] and the effectiveness of interventions such as cervical cerclage and progesterone to improve neonatal outcome remain to be proven. This may, in part, reflect an ineffective understanding of the cellular mechanisms involved and mis-categorization of patient risk status resulting in inappropriate treatment.

Mounting evidence implicates involvement of inflammatory mediators in preterm labor [12]–[13], although the processes involved are unclear. Ascending infection, it is proposed, leads to activation of inflammatory pathways, which precede cervical shortening [14] and, because of this, inflammatory mediators, including cytokines and chemokines, have been frequently measured in cervico-vaginal secretions and amniotic fluid of women delivering preterm [15]–[21]. In the most recent systematic review [22], IL-6 has been shown to be strongly associated with SPTL in asymptomatic women, whilst cervical mucus IL-8 has been used to determine if cerclage insertion will be beneficial [23]. Prospective longitudinal data which enable interrogation of temporal relationships are, however, scarce and most studies rely on a ‘snap-shot’ approach with single point sampling in the second trimester [21], [24]–[27]. It has been hypothesized, by us and others [2], [5], that inflammation precedes and mediates cervical shortening and anti-inflammatory treatments (such as progesterone) reduce further shortening as a mechanism for preventing preterm labor. Our aim was to prospectively investigate cervico-vaginal fluid inflammatory markers longitudinally in tandem with cervical length and to examine the influence of cervical cerclage and progesterone treatment. We anticipated that inflammatory responses may be specific to individual women and that there is a need to understand this process as the first step in directing interventions and informing personalized healthcare.

## Methods

### Study Population and Design

This prospective observational study was approved by St Thomas’ Hospital Research Ethics Committee (06/Q0704/66) and all patients provided written informed consent. Women were enrolled from two preterm surveillance clinics at two teaching hospitals in London between 16 and 24 weeks’ gestation from June 2006 until November 2008. Women with at least one prior spontaneous preterm birth between 16 and 34 weeks’ gestation were eligible to participate. Exclusion criteria included multiple pregnancies, previous iatrogenic preterm births and inability to give informed consent. Recruits were initially assessed every 2 weeks by transvaginal cervical length assessment as well as cervico-vaginal fluid and blood sampling between 16 and 28 weeks’ gestation. All women provided a cervico-vaginal fluid sample prior to cervical length assessment at every visit.

If the cervical length shortened to less than 25 mm before 24 weeks’ gestation, women (allocated to the case group) were offered a routine treatment (either cervical cerclage or vaginal progesterone) as per clinical practice. However, in order to ensure equal numbers for analysis and to explore the impact of treatments on cervical inflammation, women were assigned equally to either treatment using computer generated open-label randomization by the study investigator. Samples and scans were repeated weekly thereafter in women found to have a short cervix. Natural progesterone Cyclogest® 400 mg once daily (Actavis UK Ltd, Devon, UK) administered vaginally was used based on current clinical practice in light of insufficient contemporary evidence of optimal route and dose of progesterone administration. Women who did not develop a short cervix by 24 weeks’ gestation were allocated to the control group. Routine screening for vaginal organisms such as bacterial vaginosis, Trichomonas and Candida was not included in the study protocol, but if women presented with suggestive symptoms, a high vaginal swab was taken and women were treated based on antimicrobial sensitivities.

### Technique of Cervical Length Assessment

Cervical length was assessed in accordance with standardised guidelines [28]; in summary, a sagittal view of the cervix was obtained with the long axis view of the echogenic endocervical mucosa along the length of the canal, allowing identification of both the internal and external os. Without causing undue pressure on the cervix with the probe, the linear distance between the external and internal os was recorded 3 times in millimeters over a minimum of 3 minutes using optimal magnification and zoom settings and the shortest one recorded. Transfundal pressure was exerted for 15 seconds and subsequent demonstration of a funnel was noted. The total closed length in all women was measured and if a cerclage was present, the closed length cranial to the cerclage were also recorded. Approximately 90% of the measurements were undertaken by one investigator (MC), while a second investigator (AS) undertook the remaining 10%. When stored cervical length measurements were examined by both observers, the technique of cervical length assessment was reproducible (n = 30). In 95% of cases, the differences between two measurements by the same observer (MC) was ≤2.0 mm and between both investigators (MC and AS), it was ≤4.0 mm.

### Sample Preparation and Longitudinal Cytokine Analysis

At each visit, a single Dacron swab was obtained from the posterior vaginal fornix in order to obtain a high vaginal sample (referred to as a cervico-vaginal fluid, CVF). At the time of speculum examination, the swab was placed in the posterior vaginal fornix for 10 seconds to achieve saturation, then transferred into 750 µl of standard phosphate-buffered saline solution containing protease inhibitor [1 protease inhibitor cocktail tablet (Complete, Roche Diagnostics GmbH, Germany) dissolved in 50 ml standard phosphate-buffered saline solution (Sigma-Aldrich Company, Ayrshire, UK)], and immediately transported on ice to the laboratory. The swab was then removed, placed in a clean tube, vortexed for 10 seconds and centrifuged (2600 g for 10 minutes, 4°C). Resultant fluid was collected and added to the fluid in the original tube. This was mixed and centrifuged for a further 10 minutes to remove cell debris. Cell-free supernatants were divided into aliquots (110 µl) and stored at −80°C until analysis.

Longitudinal inflammatory marker analysis was undertaken on 477 individual samples from 78 women. The women included for analysis from the control group were chosen based on them having at least 4 longitudinal samples taken over the second trimester available for analysis. A minimum of six samples were analyzed for women who developed a short cervix (cases). The samples from cases included pre and post intervention samples and the sample obtained at the visit when the cervix was found to be short prior to being randomised to an intervention. Samples were thawed at room temperature, briefly vortexed and analyzed by 11-plex fluid-phase immunoassay for interleukin (IL)-1β, IL-4, IL-6, IL-7, granulocyte colony-stimulating factor (G-CSF), granulocyte-macrophage colony-stimulating factor (GM-CSF), interferon (IFN)-γ, monocyte chemotactic protein (MCP)-1, macrophage inflammatory protein (MIP)-1β and tumour necrosis factor (TNF)-α (Bio-Rad Laboratories, Hemel Hempstead, Hertsfordshire, UK) in duplicate according to manufacturer’s instructions on a Luminex-100 cytometer (Luminex Corporation, Austin, Texas) equipped with StartStation software (Version 2.0; Applied Cytometry Systems, Dinnington, UK). The choice of analytes reflected those recognized to be representative of inflammatory processes in the published literature [29] and by the investigators. A pooled control sample achieved by combining a random set of 40 samples was included in individual plates to control for inter-plate variation.

Concentrations in pg/ml were calculated from the standard curves using 4 parameter logistic regression with Bio-Plex software (Bio-Rad Laboratories, Hemel Hempstead, Hertsfordshire, UK). The lower limits of detection was 0.8 pg/ml for IL-1β, 0.5 pg/ml for IL-4, 1.1 pg/ml for IL-6, 0.5 pg/ml for IL-7, 1.1 pg/ml for G-CSF, 4.5 pg/ml for GM-CSF, 19.3 pg/ml for IFN-γ, 6.7 pg/ml for MCP-1, 1.1 pg/ml for MIP-1β and 3.0 pg/ml for TNF-α.

### Statistical Analysis

The sample size was not pre-determined due to inadequate published data informing the gestational profile of cervico-vaginal inflammatory markers, or of any relationship with repeated measures of cervical length. This study and analysis was therefore exploratory in nature. The predefined endpoints were cytokine concentrations prior to cervical shortening, and before and after treatment. Cytokine levels were expressed as pg/ml. The study was not designed or powered to directly compare the two treatment groups (e.g. for cytokine concentrations, cervical length or preterm birth), although some exploratory comparisons have been included. The data were analyzed using Stata (version 10.1, Stata Corp, College Station, Texas). Distributions of data were first established by examination of distributional plots for raw and transformed values. Log transformations were used for all biomarkers to achieve approximate normality. Estimates and tests of differences, both between and within subjects, used linear regression adjusting for repeated measures and non-normality through the use of generalized estimating equations. Where sample concentrations were below the limit of detection for the assay, an interval regression method was used, with the missing values taken as being at an unknown point on the interval between zero and the smallest positive concentration observed. The percent of samples with concentrations above the detectable range is shown in Table 1.

**Table 1:** Percentages of cervico-vaginal fluid samples with cytokine concentrations above the limit of detection for each biomarker for cases and controls between 16 and 24 weeks of gestation.

Cytokine		DetectableConcentrations %
**IL-1β**	Controls	100
	Cases	100
**IL-4**	Controls	59–78
	Cases	61–100
**IL-6**	Controls	71–94
	Cases	89–100
**IL-7**	Controls	33–58
	Cases	25–78
**IL-8**	Controls	100
	Cases	100
**G-CSF**	Controls	88–100
	Cases	100
**GM-CSF**	Controls	31–50
	Cases	39–75
**IFN-γ**	Controls	53–83
	Cases	72–85
**MCP-1**	Controls	53–78
	Cases	77–100
**MIP-1β**	Controls	88–97
	Cases	94–100
**TNF-α**	Controls	77–100
	Cases	89–100

Samples with values below the limit of detection were included in all data analysis using interval regression (see methods). Interleukin (IL)-1β, IL-4, IL-6, IL-7, granulocyte colony-stimulating factor (G-CSF), granulocyte-macrophage colony-stimulating factor (GM-CSF), interferon (IFN)-γ, monocyte chemotactic protein (MCP)-1, macrophage inflammatory protein (MIP)-1β, tumour necrosis factor (TNF)-α.

In order to determine the difference in cytokine expression in cases and controls prior to treatment, samples up to and including the visit at which the cervix shortened but prior to treatment over a period of time from 16 to 24 weeks’ were included and the average difference between cases and controls determined using a correction for effect of gestation (2-weekly categories) and inter-assay plate variation. Results were expressed as ratios of the cytokines concentrations in cases and controls with 95% confidence intervals. A p value of less than 0.05 was considered to indicate statistical significance. Actual p-values are given (usually to 2 decimal places), except for very small values, shown as p<0.001.

## Results

**Figure 1:**
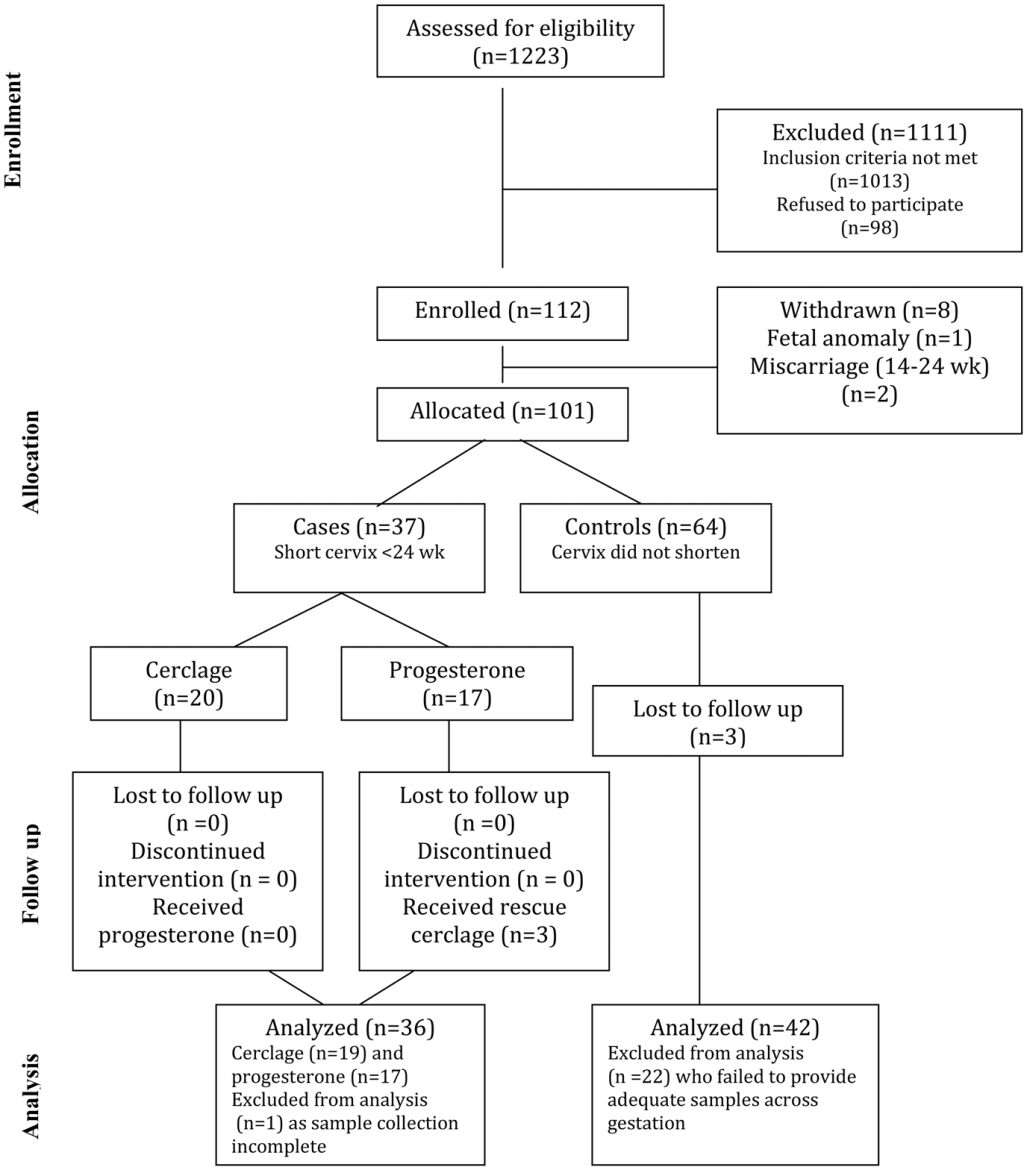
Enrolment, randomization and follow-up of participants in the study.

**Figure 2:**
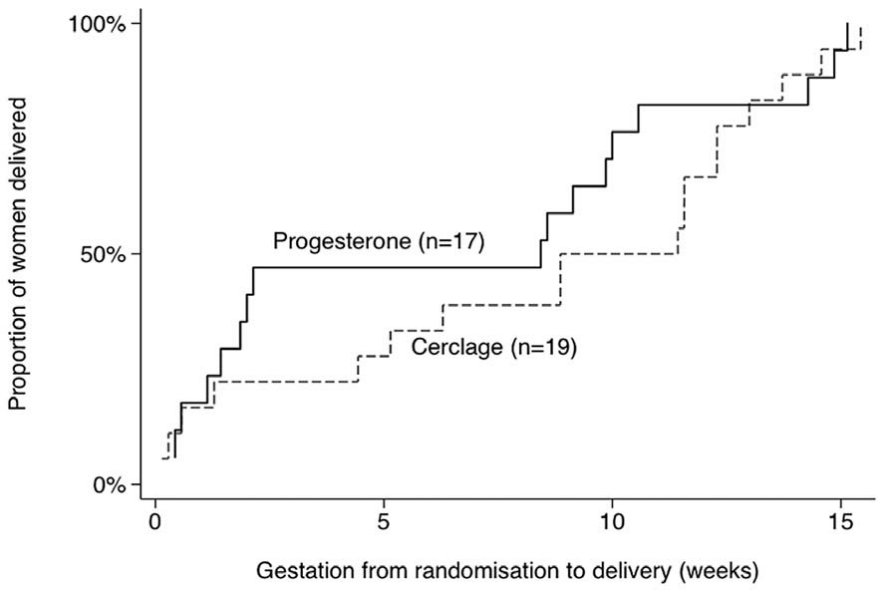
Kaplan-Meier plot to compare time to delivery following allocation to treatment with progesterone (solid line, n = 17) and cervical cerclage (dashed line, n = 19); p = 0.23. Vaginal progesterone was prescribed on the day of allocation; women with cerclage received treatment within 2.5 (SD 3.9) days.

A total of 1223 women were assessed for eligibility and 112 women were enrolled into this observational study (Figure 1). The final study group (women who provided suitable samples for longitudinal analysis of CVF cytokines) were n = 42 controls and n = 36 cases. Table 2 summarizes the baseline characteristics of the cohort providing longitudinal samples for cytokine analysis (n = 78). Women in the control group had numerically more previous preterm deliveries between 24 and 34 weeks’ gestation and were more likely to be White compared to women destined to develop a short cervix (<25 mm at <24 weeks’ gestation), who reported more previous second trimester losses and were more likely to be Black. The percentage of women with bacterial vaginosis in each group was similar (Table 2). Mean gestational age at birth in the control women was 37.9 (SD 3.49) weeks. There was no statistical difference in gestational age at birth between treatment groups (p = 0.23), with a mean gestational age at birth in the cerclage group of 33.7 (SD 7.7) weeks and 31.5 (SD 9.0) weeks in the progesterone group (Figure 2).

**Table 2:** Baseline maternal characteristics of women who had inflammatory marker analysis.

	Controls (n = 42)	Cases (n = 36)	Difference (CI)
**Maternal age (y)**	30.5 (5.9)	29.8 (6.6)	0.7 (−2.1 to 3.5)
**Height (m)**	1.6 (0.12)	1.6 (0.12)	0.0 (−0.05 to 0.05)
**Weight (kg)**	76.0 (13.5)	76.3 (17.6)	−0.30 (−7.5 to 6.9)
**Body mass index at booking (kg/m^2^)**	27.3 (6.0)	26.6 (6.2)	0.7 (−2.1 to 3.5)
**Risk factors** Previous preterm birth (24–34 weeks) Previous preterm pre-labour rupture of membranesPrevious 2^nd^ trimester loss (16–24 weeks)	23 (55%)23 (55%)20 (48%)	11 (31%)18 (50%)27 (75%)	24 (3 to 45)5 (−17 to.27)2 (−9 to 12)
**Current smoker**	3 (7%)	2 (6%)	–
**Ethnic group** * White Black Other	14 (33%) 25 (60%) 3 (7%)	4 (11%) 30 (83%) 2 (6%)	Reference 24 (6 to 43) 16 (−26 to 58)
**Positive for bacterial vaginosis if tested**	6/18 (33%)	10/31 (32%)	1 (−26 to 28)

Data are mean (standard deviation) or n (%).

* Ethnic group was self-reported.

**Figure 3:**
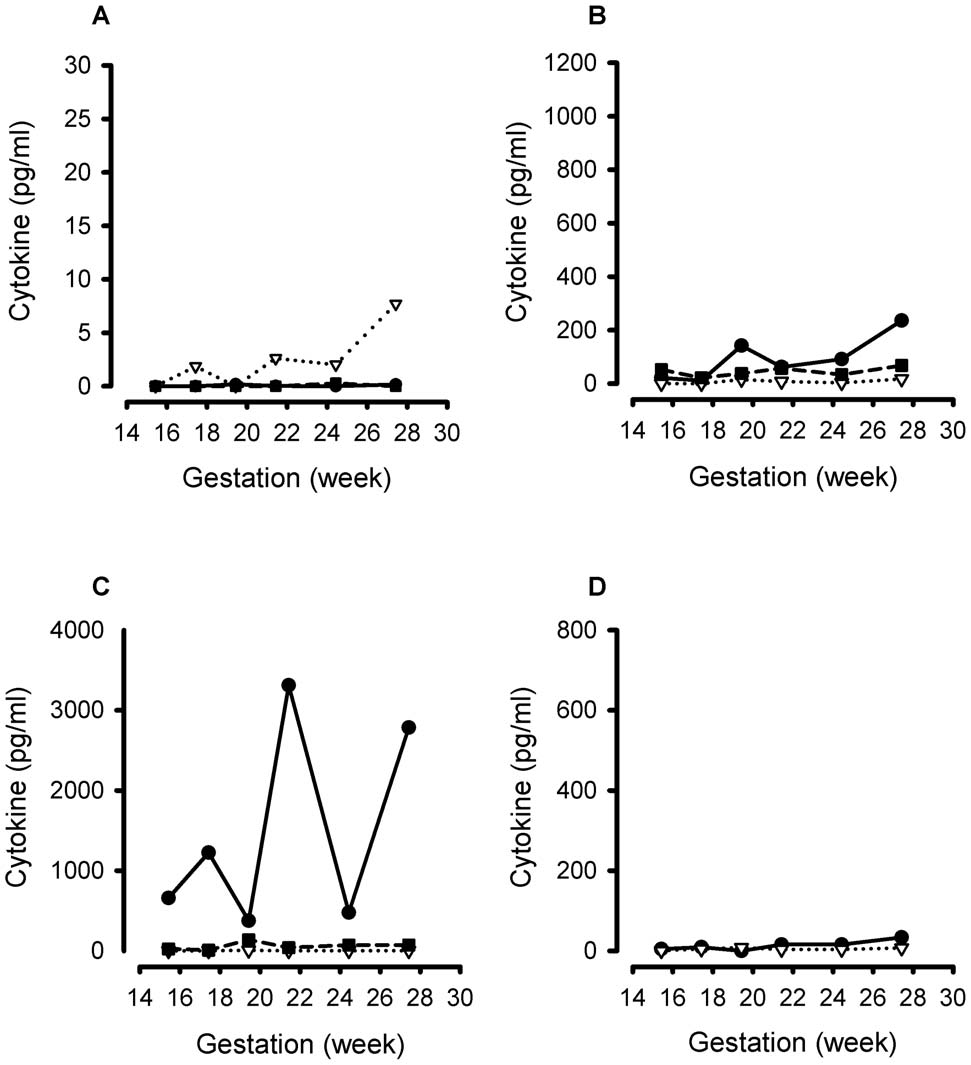
Representative gestational profile of 11 cytokines measured longitudinally in cervico-vaginal fluid samples from a pregnant woman at risk of spontaneous preterm labour who did not demonstrate cervical shortening or deliver preterm. A. Interleukin (IL)-4 (solid line, closed circle); IL-7 (dotted line, open triangle); granulocyte-macrophage colony-stimulating factor (GM-CSF) (dashed line, closed square). B. IL-1β (solid line, closed circle); interferon (IFN)-γ (dotted line, open triangle); macrophage inflammatory protein (MIP)-1β (dashed line, closed square). C. IL-8 (solid line, closed circle); IL-6 (dotted line, open triangle) circle); granulocyte colony-stimulating factor (G-CSF) (dashed line, closed square). D. Monocyte chemotactic protein (MCP)-1 (solid line, closed circle) and tumour necrosis factor (TNF)-α (dotted line, open triangle).

**Figure 4:**
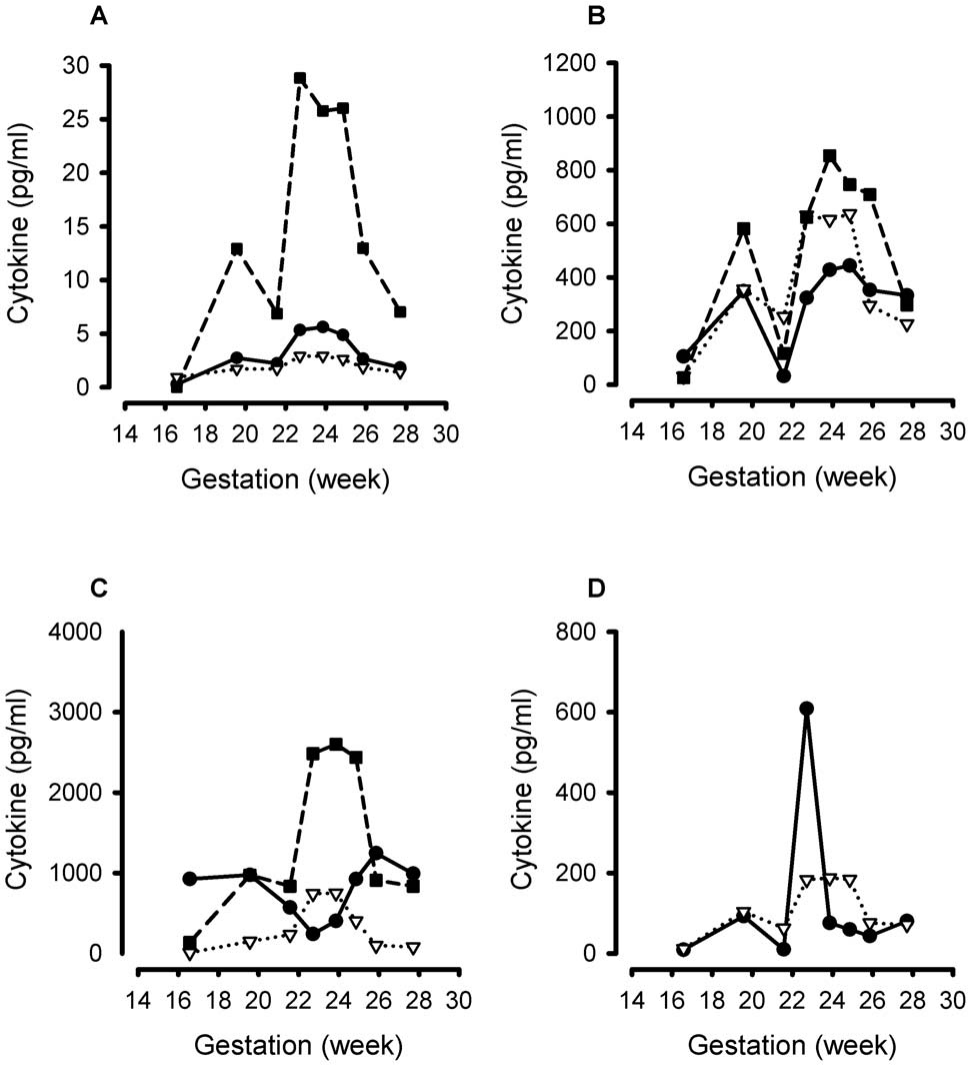
Representative gestational profile of 11 cytokines measured longitudinally in cervico-vaginal fluid samples from a representative woman at risk of spontaneous preterm labor who demonstrated cervical shortening and randomised to receive a cervical cerclage at 21^+4^ weeks’ gestation and delivered preterm at 32 weeks’ gestation. A. Interleukin (IL)-4 (solid line, closed circle); IL-7 (dotted line, open triangle); granulocyte-macrophage colony-stimulating factor (GM-CSF) (dashed line, closed square). B. IL-1β (solid line, closed circle); interferon (IFN)-γ (dotted line, open triangle); macrophage inflammatory protein (MIP)-1β (dashed line, closed square). C. IL-8 (solid line, closed circle); IL-6 (dotted line, open triangle) circle); granulocyte colony-stimulating factor (G-CSF) (dashed line, closed square). D. Monocyte chemotactic protein (MCP)-1 (solid line, closed circle) and tumour necrosis factor (TNF)-α (dotted line, open triangle).

**Figure 5:**
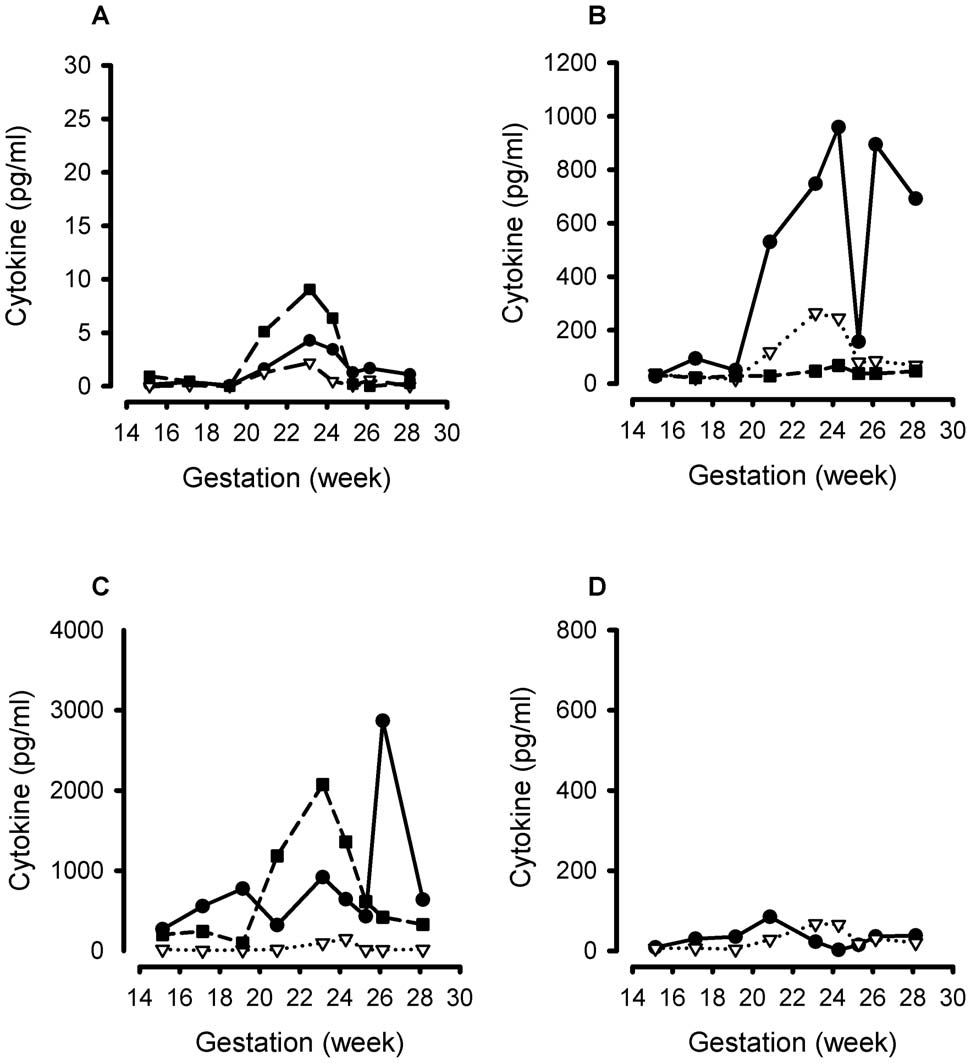
Gestational profiles of 11 cytokines measured longitudinally in cervico-vaginal fluid samples from a representative woman at risk of spontaneous preterm labor who demonstrated cervical shortening, was allocated to receive vaginal progesterone after the 23^+1^ weeks’ gestation CVF sample was taken, and who delivered preterm at 30^+1^ weeks’ gestation. A. Interleukin (IL)-4 (solid line, closed circle); IL-7 (dotted line, open triangle); granulocyte-macrophage colony-stimulating factor (GM-CSF) (dashed line, closed square). B. IL-1β (solid line, closed circle); interferon (IFN)-γ (dotted line, open triangle); macrophage inflammatory protein (MIP)-1β (dashed line, closed square). C. IL-8 (solid line, closed circle); IL-6 (dotted line, open triangle) circle); granulocyte colony-stimulating factor (G-CSF) (dashed line, closed square). D. Monocyte chemotactic protein (MCP)-1 (solid line, closed circle) and tumour necrosis factor (TNF)-α (dotted line, open triangle).

The multiplex immunoassays detected all the different inflammatory cytokines in the panel. Representative cytokine gestational profiles from 3 women are shown: a control (Figure 3), a case who received a cervical cerclage following cervical shortening (Figure 4) and a case who received vaginal progesterone following cervical shortening (Figure 5).

Overall, there was no global rise in all inflammatory markers prior to cervical shortening. However, GM-CSF and MCP-1 were raised in cervico-vaginal fluid in samples taken up to (and including) when the cervix was found to be short but prior to treatment in women at risk of SPTL (Table 3). These cytokines were raised across the 16–24 week period; there was no evidence of a progressive rise with gestation, nor an increase immediately preceding cervical shortening.

**Table 3:** Inflammation as a marker for cervical shortening.

Cytokine	Geometric Means (IU/L, 95% Confidence Interval)		Ratio (95% CI)	p value
	Control group (≤ 24 weeks’)	Short cervix group (prior to cervical shortening)		
**IL-1β**	108 (63.8 to 183)	112 (63.4 to 200)	1.04 (0.49–2.22)	0.91
**IL-4**	0.139 (0.049 to 0.391)	0.138 (0.048 to 0.392)	0.992 (0.245 to 4.01)	0.99
**IL-6**	3.37 (1.45 to 7.84)	4.88 (1.95 to 12.3)	1.45 (0.448 to 4.69)	0.54
**IL-7**	0.00850 (0.00237 to 0.0305)	0.0248 (0.006 to 0.101)	2.91 (0.546 to 15.6)	0.21
**IL-8**	528 (356 to 782)	547 (354 to 844)	1.036 (0.595 to 1.80)	0.9
**G-CSF**	84.3 (42.5 to 167)	77.8 (37.4 to 162)	0.92 (0.357 to 2.39)	0.87
**GM-CSF**	0.00423 (0.00084 to.0214)	0.068 (0.0108 to 0.435)	16.2 (1.78 to 147)	0.013
**IFN-γ**	4.63 (1.16 to 18.4)	5.19 (1.09 to 24.7)	1.12 (0.156 to 8.09)	0.91
**MCP-1**	0.871 (0.279 to 2.72)	4.21 (1.21 to 14.6)	4.83 (1.02 to 23.0)	0.048
**MIP-1β**	28.4 (18.1 to 44.7)	55.1 (31.2 to 97.0)	1.94 (0.961 to 3.91)	0.065
**TNF-α**	8.29 (4.35 to 15.8)	7.75 (3.69 to 16.3)	0.93 (0.364 to 2.40)	0.88

Comparison of eleven cervico-vaginal fluid cytokine concentrations up to 24 weeks of gestation in controls (women with cervical measurements ≥25 mm, n = 42) and cases (women who develop a short cervix, including data up until the day a short cervix was detected, but prior to receiving randomised treatment; n = 36). Ratios of geometric means are shown.

Estimates are adjusted for systematic changes with gestation and (using a pooled assay) for differences between individual assay plates. A random effects estimator is used to allow for multiple measurements per woman.

Interleukin (IL)-1β, IL-4, IL-6, IL-7, granulocyte colony-stimulating factor (G-CSF), granulocyte-macrophage colony-stimulating factor (GM-CSF), interferon (IFN)-γ, monocyte chemotactic protein (MCP)-1, macrophage inflammatory protein (MIP)-1β, tumour necrosis factor (TNF)-α.

Among the cases, mean estimates of GM-CSF [pg/ml (CI)] at 4 weeks, 2 weeks pre cervical shortening, and at cervical shortening (approximately 20 weeks on average) were 0.0015 (0.000013 to 0.18); 0.27 (0.022 to 3.3) and 0.13 (0.019 to 0.82), respectively. Corresponding values in the controls at 16, 18, 20 weeks were 0.019 (CI 0.0015 to 0.23), 0.0095 (0.0012 to 0.075), 0.0064 (0.0013 to 0.032). There was a substantial change apparent in the GM-CSF group prior to cervical shortening that did not match any change with gestation in the controls. The same pattern was found for MCP-1 [pg/ml (CI)]; corresponding values were 1.2 pg/ml (0.065 to 23), 5.6 (0.91 to 34) and 4.9 (1.3 to 19) for the cases; and 1.2 (0.17 to 8.8), 0.99 (0.21 to 4.6) and 0.65 (0.20 to 2.1) for the controls. Overall mean estimates of GM-CSF concentrations were relatively low as 82 (34%) of the 238 GM-CSF values measured up to the point of cervical shortening prior to treatment were below the lower limit of detection. These values were treated, using interval regression, as being at an unknown point in the range 0 to 0.1. GM-CSF was more often above the limit of detection in case samples than controls (Table 2). As a sensitivity analysis, a logistic regression was carried out, comparing samples with undetectable and detectable levels of GM-CSF, using the same samples as in Table 3. The odds ratio was 8.8 (1.6 to 49), p = 0.012; confirming that detectable levels of GM-CSF are linked to cervical shortening.

Vaginal progesterone treatment following cervical shortening did not significantly reduce any cytokine concentration (Table 4). Indeed, IL-1β, IL-4 and MIP-1β concentrations were higher post treatment. The insertion of a cervical cerclage was also associated with higher cervico-vaginal fluid concentrations of IL-6, IL-8, G-CSF, IFN-γ and TNF-α compared with pre-treatment values. Cerclage-associated increases in IL-1β, IL-6, G-CSF and IFN-γ were significantly greater than in the progesterone group [weekly increase of IL-1β and IL-7 concentration 15% (p = 0.02) and 63% (p = 0.02), respectively].

**Table 4:** Comparison of cervico-vaginal (CVF) cytokine concentrations before and after treatment with cervical cerclage and vaginal progesterone.

Inflammatory Marker	Cerclage Ratio (95% ConfidenceInterval; p value)	Progesterone Ratio (95% Confidence Interval; p value)	Cerclage versus Progesterone Ratio(95% Confidence Interval; p value)
**IL-1β**	3.1 (2.0–4.7; <0.001)	1.6 (1.0–2.5; 0.04)	1.9 (1.1–3.4; 0.02)
**IL-4**	2.8 (1.2–6.3; 0.02)	2.5 (1.0–6.2; 0.04)	1.1 (0.4–3.4; 0.88)
**IL-6**	5.8 (2.3–14.5; <0.001)	1.2 (0.5–3.2; 0.68)	4.7 (1.4–16.4; 0.01)
**IL-7**	1.7 (0.4–7.0; 0.49)	0.9 (0.2–4.4; 0.92)	1.8 (0.3–12.9; 0.56)
**Il-8**	1.9 (1.2–3.2; 0.01)	1.6 (0.9–2.7; 0.10)	1.2 (0.6–2.5; 0.54)
**G-CSF**	3.5 (1.7–7.4; 0.001)	0.8 (0.4–1.8; 0.56)	4.4 (1.6–12.3; 0.004)
**GM-CSF**	1.2 (0.2–6.2; 0.82)	0.3 (0.1–2.1; 0.24)	3.6 (0.4–34.9; 0.27)
**IFN-γ**	10.4 (3.0–35.9;<0.001)	1.2 (0.3–4.4; 0.80)	8.8 (1.7–47.2; 0.01)
**MCP-1**	1.7 (0.5–5.9; 0.44)	2.1 (0.5–8.1; 0.28)	0.8 (0.1–4.4; 0.78)
**MIP-1β**	2.1 (1.3–3.4; 0.001)	1.8 (1.1–2.9; 0.02)	1.2 (0.6–2.2; 0.59)
**TNF-α**	3.7 (2.1–6.7; <0.001)	1.7 (0.9–3.2; 0.08)	2.2 (1.0–4.7; 0.06)

Interleukin (IL)-1β, IL-4, IL-6, IL-7, granulocyte colony-stimulating factor (G-CSF), granulocyte-macrophage colony-stimulating factor (GM-CSF), interferon (IFN)-γ, monocyte chemotactic protein (MCP)-1, macrophage inflammatory protein (MIP)-1β, tumour necrosis factor (TNF)-α.

**Figure 6:**
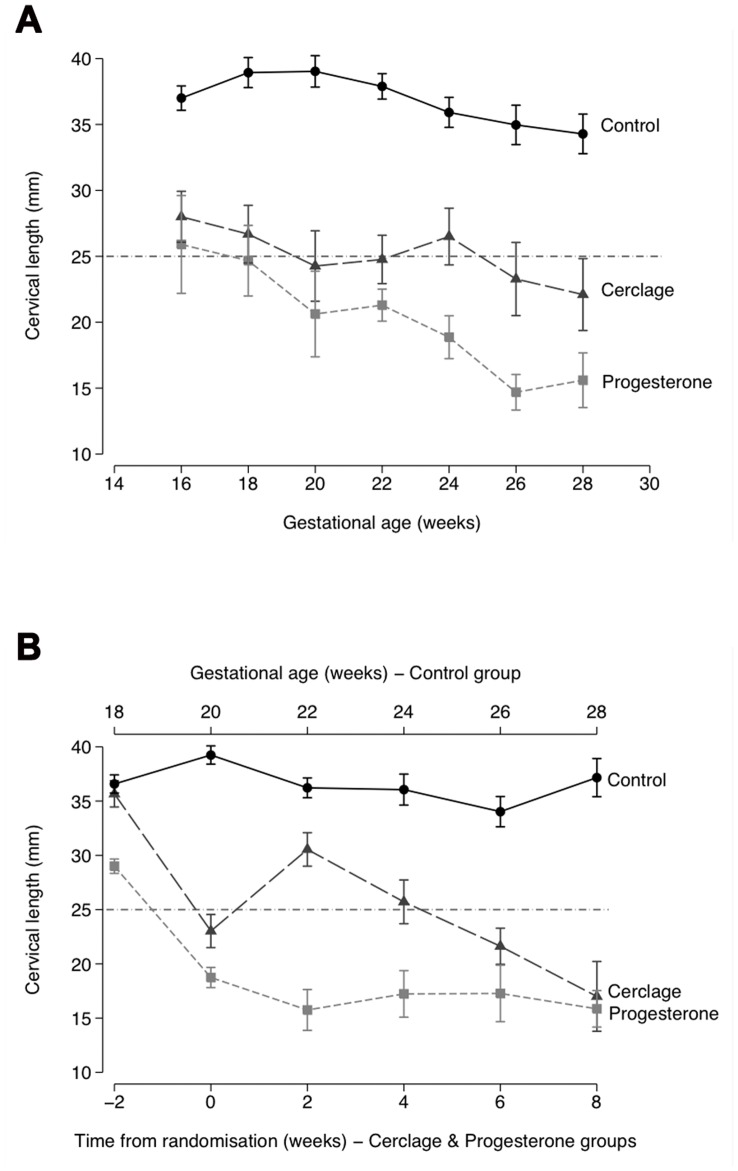
Comparison of cervical length, measured by transvaginal ultrasound *in vivo*, in controls and in women with a short cervix who were treated with a cervical cerclage or progesterone when the cervix shortened to ≤ 25 mm. A. Cervical length measurements versus the gestation at which measurements were obtained (controls, solid line, closed circles; cerclage group, dashed line, closed triangle and vaginal progesterone, dashed line, closed circle). B. Cervical length measurements versus the time from when cervical shortening was first detected (cerclage group, dashed line, closed triangle and vaginal progesterone, dashed line, closed circle). Cervical length measurements for controls are shown against gestational age (solid line, closed circles).

Cervical length at recruitment was similar in women who later received cerclage or progesterone (Figure 6A). The mean (SD) gestation at which cervical shortening was detected was 18.7 (2.5) weeks in the cerclage group [cervical length was 17.5 (6.6) mm] and 19.7 (2.8) weeks for the progesterone group [cervical length 17.0 (4.7)]. Figure 6B shows cervical length by time before and after cervical shortening by treatment group and by gestational age for controls who did not experience cervical shortening. As there was a chance difference in cervical length measured 2 weeks prior to shortening between the two groups, this was adjusted for in the analysis. Following cerclage, cervical length increased [mean increase 11.5 mm two weeks’ post-treatment (95% CI 5.9 to 17.1; p<0.001); Figure 6B]. Following progesterone treatment, cervical length did not alter significantly (Figure 6B). The mean (CI) cervical length of women in the cerclage group was 14.66 (8.42 to 20.88) mm longer than that of the progesterone group at 2 weeks post treatment (Figure 6B).

## Discussion

This study provides comprehensive longitudinal cervico-vaginal cytokine profiles of high-risk women from 16 weeks of pregnancy together with cervical length measurement before and after treatment with cervical cerclage and vaginal progesterone. The working hypothesis was based on the proposed causal relationship between inflammation and a sonographic short cervix [14]. Despite the large body of evidence to suggest that a global rise in cytokines is central to the initiation of inflammation-induced spontaneous preterm labour [15]–[16], we only detected increased concentrations of 2 ‘early inflammatory response’ cytokines, GM-CSF and MCP-1, in cervico-vaginal fluid of women destined to develop a short cervix compared to controls.

GM-CSF is a growth factor for haematopoietic progenitor cells and differentiation factor for granulocytic and monocytic cell lineages, capable of phagocytosis, antigen presentation, cytokine production and extracellular matrix remodelling [30]. Concentrations of GM-CSF are known to decrease from early- to mid-pregnancy in maternal plasma in women who have term deliveries [24], but the role of this cervico-vaginal cytokine in women at risk of spontaneous preterm labour has not been previously described. MCP-1 is a soluble chemotactic factor capable of attracting macrophages, T lymphocytes and dendritic cells to sites of tissue injury, and has been implicated in the premature activation of a cascade of inflammatory events leading to preterm delivery [31]. Cytokine analysis from umbilical cord blood taken at delivery also indicates the strong correlation between increased levels of MCP-1 and preterm delivery [32]. Our study indicates that both MCP-1 and GM-CSF cytokines may be involved in the pathophysiological process of cervical shortening early in pregnancy although concentrations in both cytokines were relatively low even in the case group. The higher concentrations of traditionally measured cytokines, IL-6, IL-1β and IL-8, in all women (including controls) suggest these cytokines play a minimal role in cervical shortening. The ethnic disparity between previous history and short cervices in our cases and controls reflects established host genotype-environment risk factors [33]–[34].

In the United Kingdom, progesterone is still not routinely administered to all women with a previous preterm delivery, but in 2011 the US Food and Drug Administration approved the use of intramuscular 17-hydroxyprogesterone caproate for prevention of preterm birth in women at risk [35]. More recently, vaginal progesterone was shown to reduce SPTL before 28, 33 and 35 weeks of gestation with improved neonatal outcome in women with a sonographic short cervix [36], [37]. However, the mechanism of action for progesterone in preventing SPTL has not been clearly elucidated. Progestational agents have been proposed to inhibit cervical ripening through inhibition of inflammatory pathways [38], thus modifying the host immune response. This hypothesis derived from animal studies [39] suggests that progesterone may prevent spontaneous preterm labour in susceptible individuals [33]. The data presented here do not demonstrate a beneficial effect of vaginal progesterone treatment on commonly measured cervico-vaginal markers of inflammation or cervical length suggesting that if this steroid does prevent early delivery, it must be acting via another mechanism. Cerclage, on the other hand, appears to cause a localized inflammatory response possibly secondary to trauma, which did not acutely induce further cervical shortening; an observation that provides evidence against the hypothesis that inflammation drives cervical shortening. The mean gestational age at delivery in women with a previous preterm birth who did not develop a short cervix was 37.9 weeks. This observation may allow clinicians to question the value of administering progesterone based on a woman’s history alone.

A strength of our study was the use of multiplex immunoassays, which allowed the measurement of several cytokines simultaneously in small volumes of cervico-vaginal fluid and thus enhanced our ability to understand the complex integrated networks in which cytokines operate [29]. Use of cervico-vaginal fluid samples also enabled us to study the local inflammatory milieu without the contribution of systemic effects on cytokine concentration which may have been secondary to other factors. A potential weakness of the study was the limited number of women who delivered preterm, although this was not our study endpoint. Whilst we could identify two biomarkers as potential predictors of a short cervix, the impact of cytokines on the preterm delivery rate could not be ascertained. Sample collection deliberately ceased at 28 weeks of gestation (when women were discharged from the preterm surveillance clinic), which precluded determination of the cervico-vaginal cytokine profiles immediately preceding labour. Given the large inter-woman variability observed, the longitudinal design allowed robust assessment of temporal changes in cytokine concentrations in each individual. Ideally, an understanding of the genetic host contribution to cytokine interaction, although outside the scope of this study, would enable further insight into the pathophysiological processes at play. Other limitations that should be considered when interpreting the findings of this study are that we did not compare cytokine profiles in high risk controls to those of a low-risk group of women recruited in parallel and that bacterial vaginosis was not routinely screened for in all recruits.

Our analysis of inflammatory markers and mediators in high-risk women provides insight into the mechanisms involved in cervical shortening and the maternal inflammatory cascade. We have highlighted that progesterone, administered vaginally at a daily dose of 400 mg, does not suppress local inflammatory events. This knowledge may not only allow a deeper insight into pathophysiology, but it may also facilitate a tailored approach to individualized healthcare, where intervention may be more appropriately targeted at those who are likely to benefit. The potential relation between GM-CSF and MCP-1 and cervical shortening will need to be validated in a larger cohort of both high and low-risk women. The relative benefit of progesterone and cerclage treatment for women at high risk of spontaneous preterm labor could be further explored in a future randomised clinical trial.
